# A bioenergetic approach favors the preservation and protection of prey, not cooking, as the drivers of early fire

**DOI:** 10.3389/fnut.2025.1585182

**Published:** 2025-05-16

**Authors:** Miki Ben-Dor, Ran Barkai

**Affiliations:** Department of Archaeology, Tel Aviv University, Tel Aviv, Israel

**Keywords:** fire, cooking, predators, human evolution, bioenergetics

## Abstract

**Introduction:**

The use of fire marks a critical milestone in human evolution, with its initial purposes debated among scholars. While cooking is often cited as the primary driver, this study proposes that meat and fat preservation, and predator protection were more likely the initial motivations for fire use by *Homo erectus* during the Lower Paleolithic (1.9–0.78 Ma).

**Methods:**

Employing a bioenergetic approach, we compared the energetic returns of hunting versus plant gathering using ethnographic data, adjusted for Lower Paleolithic conditions. Caloric content of East African prey was calculated to assess consumption duration. Archeological evidence from early fire sites was analyzed for associations with large fauna.

**Results:**

Hunting large prey (>100 kg) yielded significantly higher energetic returns (16,269 ca/h) than plant gathering (1,443 ca/h), with megaherbivores like hippopotamus providing sustenance for up to 22 days for a group of 25. Early fire sites consistently contained large fauna remains, suggesting prolonged prey consumption. Cooking offered modest energetic gains (e.g., ~1,200 ca/h for meat), insufficient to offset fire maintenance costs, unlike preservation and protection.

**Discussion:**

The substantial energetic disparity supports hunting as a dominant subsistence strategy, with fire enhancing efficiency by preserving meat and deterring predators. The prevalence of megaherbivores in Lower Paleolithic sites and heightened predation risks underscore these priorities over cooking, which likely emerged as a secondary benefit. Ethnographic analogies underrepresent these dynamics due to megafaunal extinctions altering the environment and prey availability.

**Conclusion:**

Meat preservation and predator protection, rather than cooking, were likely the primary drivers of early fire use, aligning with *Homo erectus*’ specialization in large prey acquisition. This reframes fire’s role in human evolution, suggesting it supported a hypercarnivorous lifestyle and potentially influenced cognitive development.

## 1 Introduction

The utilization of fire stands as a pivotal technological advancement in human history. Some scholars argue that rather than a sudden discovery, the mastery of fire’s control and application for human needs occurred gradually, with its origins dating back to the early Pleistocene (1–3). Substantial evidence supports the early use of fire by our ancestors during Lower Paleolithic times, starting about 1 million years before the present (4–9), although opposing perspectives exists (10). The significance of fire use in reconstructing early human adaptation strategies and capabilities holds paramount importance within Pleistocene archeology.

Wrangham, Jones (11, 12) hypothesized that the control of fire for cooking plants and meat was a driving force behind crucial adaptations in *Homo erectus*, including a reduction in digestive system size and an increase in brain size. This hypothesis, known as ‘the cooking hypothesis,’ has been a subject of debate and inquiry (2, 13–17).

The findings by Henry (18), and Henry, Büdel (19) that in some circumstances, the production of fire incurs higher bioenergetic costs than the benefits of cooking encourage reinvestigation of the reason for the use of fire by early to middle Lower Paleolithic humans. Assuming that fire was produced and maintained in the Lower Paleolithic, there is a need to consider alternative utilities or set of utilities that have rendered the acquisition and maintenance of fire economically profitable.

We employ a bioenergetic approach to explore the relative likelihood of cooking, meat and fat preservation, and protection from predators as drivers for the early use of fire. Based on accepted evidence of fire in archeological sites, we define the early use of fire as spanning from 1.9 to 0.78 million years ago (Ma), with relevance to *Homo erectus (sensu lato)*.

## 2 Materials and methods

The need to preserve and protect acquired prey arises when it is large enough to provide food for the group for several days. The extent of human involvement in acquiring large prey remains a subject of ongoing debate within the scholarly community [e.g., (20, 21)]. However, for this study, it suffices to acknowledge that early humans, specifically *Homo erectus*, did consume meat and fat of large prey. Lower Paleolithic sites are notably characterized by a substantial presence of megaherbivores, and other large animals’ remains, some of which exhibit cut marks as well as other evidence of human manipulation [e.g., (22–24)]. Smaller animal taxa were also found at Lower Paleolithic sites. However, it was demonstrated that the caloric contribution of megaherbivores was unprecedented [e.g., (25, 26)].

The relevance of ethnographic data to Lower Paleolithic circumstances is contested (27–29). In many cases, the basic preconditions for analogy of similarity in technological and environmental circumstances are unmet, specifically as they relate to large herbivores exploitation (30).

To arrive at relative energetic returns of gathering versus hunting, we used only the closest analogous available ethnographic data to the early to middle Lower Paleolithic circumstances. We based our calculations on data assembled by Kelly (31) (Table 4–5) and Morin, Bird (32) (Supplementary ESM4).

Kelly (31) dataset comprises 105 data points detailing energetic returns on plant gathering and 25 data points of hunting medium-sized prey as no data on large prey exists. Notably, the dataset does not specify the weight of the animals in question. In our analysis, we classified medium-sized animals as roughly equivalent to the size of sheep and larger.

Morin, Bird (32) dataset encompassed 129 data points. It encompasses returns derived from various hunting methods, including using guns, dogs, traps, and bow/spear, across multiple biomes, including the rainforest biome. It encompasses records of hunting a diverse range of fauna, from birds, reptiles, and rodents to larger prey. We computed average returns to ensure our results align as closely as possible with the early to middle Lower Paleolithic context. In Morin, Bird (32) dataset, hunting with firearms yielded returns above the average, while hunting in rainforests yielded returns much below the average, showing that technology and environment are substantial factors in generating energetic returns. Consequently, we calculated the average returns within Morin, Bird (32) dataset as follows:

1. The average return of the dataset (*N* = 129)
2. The average return on hunting with bows/spears (*N* = 38)
3. The average return on hunting prey, larger than 100 kgs in a non-rainforest biome with bows and spears (*N* = 8). We consider this sample to be the closest to Lower Paleolithic conditions.

To ascertain the period of consumption that prey provides to a typical group of 25 individuals (33), we calculated the caloric content of typical East African prey animals. The raw data comes from Ledger (34), who dissected 252 East African herbivores of 16 species.

In the computation of averages within category 3 (prey >100 kg, non-rainforest), sourced from the Morin, Bird (32) table, we deliberately excluded prey weighing less than 100 kilograms. This exclusion is justified because prey in the 100-kilogram range typically contains approximately 60,000 calories, which a group of hunter-gatherers can reasonably consume in a single day. In stark contrast, a single 1.5-ton Hippopotamus, yielding about a million calories, can sustain such a group for 22 days (Table 1). Consequently, the relative economic significance of protection from predators and preservation in the context of medium-small prey would have been considerably lower, compared to larger prey.

**Table 1:** Caloric content of selected East African prey [based on data in (34); see Supplementary material].

Common name	Scientific name	Body weight (kg)	Caloric content (kilocalories)	Days of consumption
Hippopotam (F)	*Hippopotamus amphibius*	1,277	1,093,457	22
Buffalo (M)	*Syncerus caffer*	753	525,022	10
Eland (M)	*Taurotragus oryx*	508	406,518	8
Oryx (F)	*Oryx beisa*	161	141,299	3
Topi (F)	*Damaliscus lunatus*	104	57,127	1
Impala (M)	*Aepyceros melampus*	57	32,539	0.5
Thomson’s Gazelle (M)	*Eudorcas thomsonii.*	25	14,741	0.3

F, Female; M, Male. Consumption days for a group of 25 people at an average of 2000 calories per day (26).

While it is true that bows were not accessible to hunters in the early to middle Lower Paleolithic, energetic return data exclusively focusing on spear employment is notably absent. Furthermore, the other categories encompassed within the hunting technology parameter, namely guns, held even less relevance in hunting large prey during the early to middle Lower Paleolithic. Mass hunting, traps, and hand digging, the remaining categories, were predominantly employed for hunting small animals in the dataset, rendering them equally irrelevant in the analogy.

## 3 Results

### 3.1 Days of storage of prey by size

To demonstrate the relative duration of the predation risk and preservation need, we calculated in Table 1 the days of subsistence that prey in Ledger (34) dataset would provide a group with 25 members (26). Early Lower Paleolithic elephants (Elephas racki) could weigh 10 tons, so several times the heaviest animal in the data set (*Hippopotamus amphibius* at 1.4 tons), so they would have provided meat and fat for a much longer period. However, the hunting of large prey motivated several contemporary hunting groups to consolidate at the hunting site (35), so by analogy, the figures might have been lower than calculated but in any case, remain high.

### 3.2 The difference between hunting and plant gathering returns

Table 2 presents the average bioenergetic return derived from plant gathering and prey hunting, quantified in calories per hour (ca/h) and the corresponding 95% confidence intervals (95% CI). It is evident from Table 2 that hunting in a rainforest biome yields a substantially lower average return of 3,342 ca/h, albeit still double that of plant gathering. Plant gathering yields an average return of 1,443 ca/h with minimal disparity in the returns from various plant foods, such as seeds and tubers, as indicated by the narrow 95% confidence interval range of 1,025–1861 ca/h. It is less than one tenth of the return of 16,269 calories per hour on hunting >100 kgs prey with bow and arrows in non-rainforest biomes. It also less than one tenth of the average of the energetic return on the whole dataset of 14,877 calories per hour.

**Table 2:** Average energetic returns (calories per hour) from plant gathering and prey hunting (31, 32).

Category	N	Average energetic return ca/h	95% CI
Kelly plants	105	1,443	1,025–1861
Kelly medium-size prey	25	19,227	13,846– 24,608
Morin et al. Total sample	129	14,877	11,138–18,615
Morin et al. Guns	35	27,007	17,694–37,425
Morin et al. Bow/spear rainforest	26	3,342	1807–4,877
Morin et al. bow/spear >100 kg	9	15,509	14,815–9,679
Morin et al. bow/spear >100 kg in non-rainforest	8	16,269	5,424–27,113

See Supplementary material for raw data.

### 3.3 The association between fire remains and large prey

In an ideal scenario, our approach would involve compiling a list of early and middle Lower Paleolithic sites featuring large fauna and examining the presence or absence of fire in these archeological contexts. However, undertaking such an endeavor would prove futile due to the significantly poorer preservation of fire evidence than faunal remains. This challenge is particularly pronounced in open-air sites, which predominantly characterize the early to middle Lower Paleolithic period, as opposed to cave sites which usually reveal better fire preservation (14, 36). Following these preservation limitations, we have assembled in Table 3 a list of all reported earliest sites (dating back to before 0.78 million years ago) where fire has been claimed to be present and for which a faunal record is available. Remarkably, our examination of these sites reveals a consistent pattern—all contain remains of very large herbivores. It should be noted that early fire does not lend itself easily to be identified, so it might be the case that other Lower Paleolithic sites bearing large mammals did contain a fire that was not recognized. Under different circumstances, as a different number of individual prey animals hunted, the size of the group, and the time spent at the site, different decisions regarding the use of fire would have been taken. We suggest that in cases where the consumption of megaherbivores was planned for a prolonged duration, the use of fire was highly likely to preserve meat and fat and to keep predators away (37). Interestingly, Bellomo (38) (p. 194) analyzed the potential use of fire in Koobi Fora FXJ20 Main and after eliminating several uses states: “It is most likely that the early hominids at FxJj 20 Main primarily used fire as a source of protection, a source of light, and/or as a source of heat.”

**Table 3:** Early and middle lower Paleolithic sites with evident traces of fire and the presence of large fauna.

Site	Age	Large Fauna	References
Wonderwerk stratum 10,11,12, S. Africa	1.0–1.96 Ma	Equidae, Large Bovidae	(4, 109)
Koobi Fora, Kenya FXJj 20 AB,	1.5 Ma	Hippopotamidae, Giraffidae, Rhinoceratidae in FwJj14A, FwJj14B, and GaJi14	(110–112)
Koobi Fora, Kenya FXJj 20 Main,	1.5 Ma		(38)
Gesher Benot Ya’acov, Israel	0.78 Ma	Elephants, Hippopotamus	(9, 60)
Swartkrans, Member 3, S. Africa	1–1.5 Ma	Elephas, Equus, Hippopotamus,	(113, 114)
Gadeb 8E, Ethiopia	1 Ma	Hippopotamus, Elephant, Equus	(115, 116)
Chesowanja, Kenya	1.42	Bovids, equids, hippopotamus	(7)
Evron Quarry, Israel	1.0–0.8 Ma	Elephant, Hippopotamus	(6, 117)
Cueva Negra, Spain	0.78–0.98 Ma	Stephanorhinus, Bison,	(5, 118)

### 3.4 The energetic return on cooking

A consensus exists in the literature that cooking enhances the bioavailability of energy from plant-based foods, such as tubers and seeds (39–42). While the denaturation of proteins and the loss of structural integrity in meat are expected to augment its energetic value when cooked, several factors, including the toughening of meat fibers, Maillard reactions, and particularly the loss of fat during field roasting, counteract these benefits. Experimental studies involving mice fed cooked and uncooked meat—designed to eliminate the variable of fat loss—have yielded inconclusive results regarding the net energetic contribution of cooked meat. Moreover, these studies have not quantified the magnitude of any such contribution (17, 40).

Bioenergetic returns for both plant-based and animal-based foods are commonly calculated based on their theoretical maximum caloric content rather than their actual digestible caloric contribution, which remains empirically undetermined. Consequently, the values presented in Table 2 represent maximal estimates. Given the low initial average bioenergetic return of 1,443 ca/h for plant-based foods and a narrow 95% confidence interval (1025–1861 ca/h), the caloric gains attributable to cooking plants are likely to be minimal compared to those for meat. Magargal (42) (Table 3) quantifies the increase in digestibility due to cooking as 14–23%, 12.7, and 3.7% for starch, protein, and lipid components of plant foods, respectively. This energetic increase equates to a modest increase in energetic return, on the order of a few hundred calories per hour. Even if these digestibility gains were doubled, they would still be insufficient to offset the caloric cost of maintaining a fire, estimated to be in the high hundreds of calories per hour (18, 19) (Table 3) or be comparable to the return benefits of preservation and predator protection of large prey. While using smaller, short-term fires for episodic cooking could potentially reduce collection costs, it is less probable that such fires would leave detectable traces hundreds of thousands of years later (43).

As for meat, the positive impact of cooking on protein digestibility (12.7%) is relevant to only half of the caloric content in large animals (44) (Table 1). The remaining caloric content is lipid-based, for which cooking enhances digestibility by a mere 3.7%. Consequently, the weighted average increase in digestibility due to cooking would contribute approximately 8%, or around 1,200 ca/h, to the overall energetic return. Consequently, energetic return for uncooked meat would approximate 15,000 ca/h (i.e., ~16,200 ca/h minus 1,200 ca/h).

The energic cost of maintaining fires is highly variable and contingent upon environmental factors, making it difficult to quantify precisely. Nevertheless, even under the assumption of low cost for cooking plants, the energetic return from uncooked meat remains over 10 times greater than that from cooked plants (~15,000 ca/h versus 1,443 ca/h). In summary, whether cooked or uncooked, the bioenergetic yield from hunting substantially surpasses that from plant gathering.

## 4 Discussion

### 4.1 Energetic return and behavior

According to the Optimal Foraging Model (OFM) (45), the pronounced disparity in energetic returns between plant gathering and prey hunting suggests a predominance of hunting in early human subsistence strategies.

The OFM model employs the relative availability of prey and plant resources to predict dietary choices based on *a priori* rankings of food items. It is thus crucial to note that the abundance of large prey was considerably higher in periods preceding the Late Quaternary, prior to the megafaunal extinction events (23, 46–48). The extinction of megaherbivores led to a significant reduction in the faunal carrying capacity of ecosystems while concurrently increasing the vegetation carrying capacity (49–52). Consequently, large prey was more readily available to early humans than in contemporary settings, and its relative abundance compared to plant resources was likely higher.

### 4.2 Large prey consumption in the lower Paleolithic

A conspicuous presence of large and very large prey in early to middle Lower Paleolithic archeological sites is very common. Recent analyses of the archeozoological and paleontological East African record portray *H. erectus* as a habitual hunter of large prey (24, 53, 54). Preference for large prey animals during the Pleistocene is a conventional interpretation of archeological assemblages [e.g., (55, 56)]. Large animals, including elephants, are a common feature in African early Pleistocene sites (55, 57–59) and sites outside Africa, such as Latame, Ubeidiya, Revadim and Gesher Benot Yaaq’ov in the Levant, Dmanisi in Georgia (25, 60–64), Marathousa in Greece (65, 66), 21 sites with *Mommuyhus meridionalis* remains in association with Acheulean or Oldowan stone tools in Spain (67), Castel de Guido and eight other elephant butchering sites in Italy (68) and Schoningen in Germany (69, 70). Indeed, according to Werdelin and Lewis (71), beginning 1.5 Mya, humans became members of the hypercarnivore guild, specializing in acquiring large herbivores, as evidenced by the extinction of sabertooth predators and some hyenas. At the same time, there was no unusual decline in the under-21 kg hypo-carnivore group. A similar phenomenon of large carnivore extinction a few hundred thousand years after humans’ arrival was recently described in Spain (72). The decline of large carnivores in conjunction with human peopling and competition was also identified in Italy (73). Large animals, including elephants, continued to be a visible component of archeological sites worldwide throughout the Pleistocene (23, 67, 68, 74–78). Stiner (79) (p. S288) writes, “… hominins were big-game hunters, and they were rather specialized in their focus on ungulate prey.”

A multidisciplinary literature review including evidence from human physiology and genetics, archeology, paleontology, and zoology found that *H. erectus* was likely a hypercarnivore, consuming over 70% of his energy from meat and fat and specializing in obtaining large prey (80). We conclude that large prey contributed significantly to humans’ bioenergetic economy during the studied period. Consequently, large prey preservation by smoking and protection must have been a significant economic need for Paleolithic hunter-gatherers.

### 4.3 Meat preservation

A prominent impetus for fire utilization could be its role in food preservation through smoking and drying, extending the shelf-life of energetically valuable resources. This fire use would render fire a more energetically efficient tool for preserving the yield of hunting activities than for cooking plants or meat. The early use of fire for meat preservation may be less controversial than for predator protection, as evidence suggests various methods of meat preservation were employed, including drying and smoking, by late Paleolithic and recent hunter-gatherers (8, 43, 81, 82). Moreover, it was demonstrated that late Lower Paleolithic humans intentionally preserved marrow within fallow-deer limb bones for several weeks while stored in a cave, thus supporting the attention paid to food preservation in the Paleolithic (83). Additionally, there is proof that humans can consume putrid meat (84), and thus, prey consumption could be extended beyond our modern standards.

The paucity of fire evidence in early archeological sites could be due to the poor preservation of campfires (14), or it may indicate that fires were primarily employed in sites characterized by intensive large prey consumption, like Gesher Benot Ya’acov where the energetic costs of wood collection would have been justified in order to preserve and protect the huge quantities of meat and fat consumed at the site (9, 25, 60). While the use of torches for active scavenging to deter predators from hunted prey (85) or to impede the mobility of megaherbivores during hunts (86) is theoretically conceivable, empirical evidence for such practices remains conspicuously absent from the archeological record.

### 4.4 Anti-predation demands on *Homo erectus*

The threat posed to *Homo erectus,* and its possession of prey would have been substantial. Interspecific killings are common among carnivores with intermediate differences in body size and comparable predatory habits (87). *Homo erectus*, with an estimated body weight of 60–70 kgs (88) and targeting large prey, meets this definition. Predatory species of the Hyaenidae family, such as the *Hyaena hyaena* and the Crocuta genus were kleptoparasites, and theft of prey was also observed in lions (89).

Mitigating predation pressure constitutes a significant facet of animal behavior, as extensively documented in the literature (90). For instance, chimpanzees employ arboreal nesting as a defensive strategy against predators (91). The transition to terrestrial sleep is generally attributed to *Homo erectus* (92). As an ancillary hypothesis, the necessity to guard large prey over extended periods by *Homo erectus* could have catalyzed this behavioral shift.

Notably, the risk profile for *Homo erectus* diverged substantially from that of other carnivores. Unlike many carnivores, humans often transported their kills to a centralized location (93). A single megaherbivore could contain calories ranging from 1 million to several million (25, 26, 94), thereby sustaining a community for an extended duration (Table 1). Consumption would span days, weeks, or even months. Gaudzinski-Windheuser, Kindler (26) (p. 11) posit that, based on the caloric content of Straight-tusked elephants at the Neumark-Nord 1 site, a local hunter-gatherer group of approximately 25 individuals could be sustained for a minimum of 3 months, contingent upon the availability of food preservation methods.

During this extended period, the partially consumed carcass would inevitably attract both predators and scavengers, thereby elevating the risk to both the prey and the human group. Disentangling the risk of prey theft from the risk of predation upon humans is both impractical and likely inconsequential. While the economic cost of theft can be quantified (Table 2), the risk to human life defies easy quantification. The presence of partially consumed megaherbivores would have intensified the predation risk, making it a more acute issue for *Homo erectus* compared to its predecessors, who generally targeted smaller prey (24).

In summary, the risk profile for *Homo erectus* extended beyond the immediate threat of loss of life to include the potential forfeiture of substantial energetic investments in procuring food. Moreover, the temporal proximity of predators shifted from intermittent episodes lasting minutes or hours to a near-constant presence, particularly if the habitual consumption of large prey constituted a significant portion of the diet. Thus, fire could provide a significant risk-reduction mean to humans in the discussed period. The partial transition to cave habitation during the Late Middle Pleistocene [e.g., (36)] mitigated predation risks by constraining the avenues of approach available to predators. While caves were frequented by apex predators such as cave lions, cave bears, and hyenas, partially consumed prey and preserved anatomical components [e.g., (83)] would have invariably attracted both predators and scavengers. Despite this, scant evidence suggests interspecific prey sharing within cave environments, possibly attributable to the use of fire [e.g., (95)].

Other uses of fire by current hunter gatherers such as warmth, ritual, raw material processing, light and protection from insect (43) cannot be ruled out but do not lend themselves to a simple economic modeling, the preferred method of investigation in this paper.

### 4.5 The relationship between prey preservation and protection hypothesis and the cooking hypothesis

We do not contend that fire was not used for cooking during the early to middle Lower Paleolithic periods and beyond. Empirical evidence indicates that fish cooking, for example, occurred at Gesher Benot Ya’akov approximately 780,000 years ago (96). Such culinary activities would not undermine our hypothesis; if a fire were available for prey preservation and protection, its multifunctional utility for cooking, warmth, illumination, and social cohesion would naturally be exploited at no additional cost. However, the assertion that *Homo erectus*’ reduced masticatory requirements and capacity for high-quality food consumption were contingent upon cooking (12) is challenging to reconcile. Zink and Lieberman (97) demonstrated that the mechanical reduction of food by stone tools into smaller fragments could diminish masticatory demands. Significant consumption of soft fat tissue by *Homo erectus* due to limited protein metabolism capabilities (25, 80, 98) could also enable a smaller masticatory system.

Contrary to Wrangham’s original hypothesis, which posits cooking as the catalyst for key evolutionary adaptations in *Homo erectus*, our hypothesis redirects the focus toward specialization in large prey hunting. Consumption of plants by hominins continued as it had for millions of years, and when cooking had taken place, it could have been done on fires that were initiated to preserve and protect prey. We have delineated additional, non-faunal evidence supporting *Homo erectus*’ specialization in large prey acquisition and describing possible adaptations that can be attributed to this specialization (44, 80). By specialization in large prey, we mean that *Homo erectus* were adapted and likely energetically dependent on the acquisition of significant quantities of large prey. There is evidence that *Homo erectus* did acquire smaller prey and consumed plants (60, 99). The cognitive demands associated with fire production and maintenance for preservation and protection could have been instrumental in driving the observed protracted increase in average brain volume in *Homo erectus* (100). This hypothesis is not so different than Wrangham’s as it set the need to produce fire as one of the drivers of *Homo erectus*’ evolution. However, it emphasizes the specialization in acquiring large prey as its driver.

### 4.6 Discrepancy between ethnography-based protection needs and energetic return data and early Paleolithic conditions

Ethnographic analogies with Paleolithic circumstances have been used to argue that, in the Paleolithic, protection from predators was a negligible requirement (43), and that the energetic return on hunting was much lower than our prediction, around 980 calories per hour (101). The validity of analogies between recent and Paleolithic hunter-gatherer behavior has been the subject of prolonged debate, sometimes leading to strong statements such as “To hell with Ethnoarchaeology” (27) and “The tyranny of ethnography” (28). Over 60 years ago, the founders of modern archeology established that any analogy between recent and later occurrence must meet the fundamental requirement of comparable environments and technologies (102). This is even more critical when seeking quantitative values like energetic returns, which are directly affected by environmental conditions and technological capabilities. We addressed this lacuna in detail in a paper dedicated to this problem (30), so we will only briefly review the main arguments here.

Foraging optimization models often use energetic returns as a key factor in determining optimal strategies. Accordingly, prey is added to the diet in descending order of energetic return (103, 104). Thus, the pronounced disparity in energetic returns between plant gathering and prey hunting suggests a predominance of hunting in early human subsistence strategies.

Another factor in food items ranking is the relative availability of prey and plant resources. It is thus crucial to note that the abundance of large prey was considerably higher in periods preceding the Late Quaternary Megafaunal Extinction and later, Holocene extinctions (23, 46–48). The extinction of megaherbivores led to a significant reduction in the faunal carrying capacity of ecosystems while concurrently increasing the vegetation carrying capacity (49–52). Consequently, large prey was more abundant compared to plant resources before the Late Quaternary, increasing its relative acquisition in previous periods, compared to ethnography-based findings.

Large herbivores also create and maintain open landscapes, which affect the relative ease of searching for and acquiring prey and plants, and the relative faunal biomass, which tends to be higher in open landscapes (105).

Kraft, Venkataraman (101) dataset which claims average energetic return of 982 cal/h is largely composed of rainforest groups who practice part-time horticulture. As we saw in Table 2, energetic returns in rainforest are some five times lower than in open forests. Moreover, their extremely low 982 calories per hour return does not align with Morin, Bird (32) returns, even when we normalize them for the search costs included in Kraft et al.’s net calculations. For example, a common Paleolithic prey, a buffalo, contains 550,000 calories (Supplementary material). Morin et al.’s average of 14,877 calories means that handling and pursuit would have taken (550,000 / 14,877) 37 h to complete. Applying Kraft et al.’s 982 calories per hour net return, including search, results in (550,000 / 982) 560 h for handling, pursuit, and search, so (560–37) 523 h for search. Assuming 8-h search days, this equates to 65 days of search before hunters were supposed to hunt one buffalo, while a buffalo could last a 25-person group for about 9 days (550,000 / (25 × 2,500)). This example clearly shows that the return on hunting could not have been that low, or there would not have been evidence for the acquisition and exploitation of buffalo (or Bos/Bison) in the Paleolithic. In other words, Kraft, Venkataraman (101) results may be correct for hunting of small prey like squirrels and rodents in a South American Jungle, where search time per calorie could be substantial but not for large highly visible prey almost nine hundred kgs bigger, which lives in large herds in the African open savannahs. On a side note, it is doubtful that humans could survive in the rainforest solely on hunting and gathering with such low returns without the relatively high return on horticulture (101).

Regarding McCauley, Collard (43) claim of little fire use for protection in recent hunter-gatherers, the effects of the extinctions of large herbivores and the resulting non-analogous present environment (48) on the need for fire for preservation and protection are substantial. The megafauna extinction also caused the extinction of large carnivores (106). The decline in large prey led to the hunting of smaller prey that lasted for less time, hence reducing the need for protection and preservation. Coupled with the presence of dogs in many recent hunter-gatherer camps, the need for fire use for protection must have declined substantially, compared to earlier periods.

In summary, McCauley, Collard (43) results and Kraft, Venkataraman (101) cannot be used as an inference to the Lower Paleolithic period.

## 5 Conclusion and future research

The study connects early fire use to dietary strategies, emphasizing the nutritional importance of meat and fat from large prey in the Lower Paleolithic. It challenges assumptions about the primacy of cooking in shaping human nutritional evolution, potentially influencing how we understand dietary adaptations in *Homo erectus.*

By focusing on bioenergetic costs and benefits, the paper reframes fire use as a strategic adaptation to maximize returns from hunting, a key subsistence activity for *Homo erectus*.

In archeology, behavioral ecology is often employed to elucidate decisions related to prey hunting and plant gathering (107).

In this context, we delved into the bioenergetic implications that sustaining fire incurs energetic costs, necessitating a net positive energetic return to justify its production and maintenance. Our hypothesis posits that the primary impetus for early fire use lay in the imperative to preserve and safeguard large prey from predators during the extended period of its consumption. We draw upon the significantly higher energetic returns associated with hunting prey than gathering plants to substantiate our hypothesis.

In recent hunter-gatherers, the average energetic yield from plant foraging is less than one-tenth of the return obtained from the most analogous hunting prey weighing over 100 kg in non-rainforest African environments (Table 2). Although we argue that ethnographic data may not serve as an ideal analogy for the Lower Paleolithic, the substantial disparity in energetic returns between plant gathering and hunting and the higher relative density of large herbivores in the earlier times suggests that a similar, sizable difference likely existed during the early to middle Lower Paleolithic. The present energetic return gap that is measured on a background of increased relative biomass of plants (106) strengthen the case for the existence of the plant animal return gap during the Paleolithic period when relative plants abundance was smaller.

The substantial evidence for the consumption of large prey by *Homo erectus* validates a need to preserve and protect prey remains for many days. The relative gain of energetic return from the prevention of prey deterioration and theft substantially outweighs the gains that cooking could provide and thus should have been a more likely driver of the production of fire. Other uses of fire like warmth and light do not lend themselves to simple economic modeling so could not be rejected or supported using our method.

Although cooking is stated as the most common use of fire among recent hunter-gatherers, we argued that the presence of dogs and of smaller prey due to the megafauna extinction throughout the Pleistocene and the Holocene have reduced the need for the preservation of prey and the protection from diminishing guilds of large predators.

Since there is little knowledge of the extent of which Early Pleistocene predators and scavengers would have been deterred by fire, the relative importance of preservation and protection in the early use of fire remains open.

The cognitive adaptations that presumably were required for fire production, combined with the need to adapt to acquire large prey, together may explain the marked increase in *Homo erectus’* brain volume. In return, large fat and meat deposits, as well as fire protection and preservation, sustainably supported the extensive caloric demands of *Homo erectus*. This hypothesis is associated with our unifying hypothesis explaining human physiological and cultural evolution as an adaptation to varying prey sizes during the Paleolithic period (44, 108).

The hypothesis could be further scrutinized by examining temporal trends in the co-occurrence of predator gnawing marks and human consumption marks on faunal remains and by contrasting gnawing rates at sites with and without fire traces. However, such testing is constrained by uncertainties surrounding the anthropogenic origins of fire at identified sites and the absence of fire at others. The scarcity of early sites with pertinent evidence further complicates empirical validation. However, the nexus between evidence of fire and the presence of large prey mammals at several early-middle Lower Paleolithic sites strongly supports the hypothesis presented here.

We hope the prey preservation and protection hypothesis will generate more relevant research and eagerly await further developments in the field.

## Data Availability

The original contributions presented in the study are included in the article/Supplementary material, further inquiries can be directed to the corresponding author/s.
